# Humoral Immune Responses after an Omicron-Adapted Booster BNT162b2 Vaccination in Patients with Lymphoid Malignancies

**DOI:** 10.3390/v16010011

**Published:** 2023-12-20

**Authors:** Line Dam Heftdal, Cecilie Bo Hansen, Sebastian Rask Hamm, Laura Pérez-Alós, Kamille Fogh, Mia Pries-Heje, Rasmus Bo Hasselbalch, Dina Leth Møller, Anne Ortved Gang, Sisse Rye Ostrowski, Ruth Frikke-Schmidt, Erik Sørensen, Linda Hilsted, Henning Bundgaard, Peter Garred, Kasper Iversen, Caroline Sabin, Susanne Dam Nielsen, Kirsten Grønbæk

**Affiliations:** 1 Viro-Immunology Research Unit, Department of Infectious Diseases, Section 8632, University of Copenhagen, Rigshospitalet, Blegdamsvej 9, 2100 Copenhagen Oe, Denmark; 2 Department of Haematology, Copenhagen University Hospital, Rigshospitalet, Blegdamsvej 9, 2100 Copenhagen Oe, Denmark; 3 Biotech Research and Innovation Centre, University of Copenhagen, Ole Maaloees Vej 5, 2200 Copenhagen N, Denmark; 4 Laboratory of Molecular Medicine, Department of Clinical Immunology, Section 7631, Rigshospitalet, Ole Maaloees Vej 26, 2200 Copenhagen N, Denmark; 5 Department of Cardiology, Copenhagen University Hospital, Herlev and Gentofte Hospital, Borgmester Ib Juuls Vej 11, 2730 Herlev, Denmark; 6 Department of Emergency Medicine, Copenhagen University Hospital, Herlev and Gentofte Hospital, Borgmester Ib Juuls Vej 11, 2730 Herlev, Denmark; 7 Department of Clinical Medicine, University of Copenhagen, Blegdamsvej 3B, 2200 Copenhagen N, Denmark; 8 Department of Cardiology, Copenhagen University Hospital, Rigshospitalet, Blegdamsvej 9, 2100 Copenhagen Oe, Denmark; 9 Department of Clinical Immunology, Section 2034, Copenhagen University Hospital, Rigshospitalet, Blegdamsvej 9, 2100 Copenhagen Oe, Denmark; 10 Department of Clinical Biochemistry, Copenhagen University Hospital, Rigshospitalet, Blegdamsvej 9, 2100 Copenhagen Oe, Denmark; 11 Centre for Clinical Research, Epidemiology, Modelling and Evaluation, Institute for Global Health, UCL, Royal Free Campus, Rowland Hill St, London NW3 2PF, UK; 12 Department of Surgical Gastroenterology and Transplantation, University of Copenhagen, Rigshospitalet, Blegdamsvej 9, 2100 Copenhagen Oe, Denmark

**Keywords:** lymphoid malignancies, COVID-19, vaccine, booster, variant, SARS-CoV-2

## Abstract

To accommodate waning COVID-19 vaccine immunity to emerging SARS-CoV-2 variants, variant-adapted mRNA vaccines have been introduced. Here, we examine serological responses to the BA.1 and BA.4-5 Omicron variant-adapted BNT162b2 COVID-19 vaccines in people with lymphoid malignancies. We included 233 patients with lymphoid malignancies (chronic lymphocytic B-cell leukemia: 73 (31.3%), lymphoma: 89 (38.2%), multiple myeloma/amyloidosis: 71 (30.5%)), who received an Omicron-adapted mRNA-based COVID-19 vaccine. IgG and neutralizing antibodies specific for the receptor-binding domain (RBD) of SARS-CoV-2 were measured using ELISA-based methods. Differences in antibody concentrations and neutralizing capacity and associations with risk factors were assessed using mixed-effects models. Over the period of vaccination with an Omicron-adapted COVID-19 vaccine, the predicted mean concentration of anti-RBD IgG increased by 0.09 log10 AU/mL/month (95% CI: 0.07; 0.11) in patients with lymphoid malignancies across diagnoses. The predicted mean neutralizing capacity increased by 0.9 percent points/month (95% CI: 0.2; 1.6). We found no associations between the increase in antibody concentration or neutralizing capacity and the variant included in the adapted vaccine. In conclusion, a discrete increase in antibody concentrations and neutralizing capacity was found over the course of Omicron-adapted vaccination in patients with lymphoid malignancies regardless of the adapted vaccine variant, indicating a beneficial effect of Omicron-adapted booster vaccination in this population.

## 1. Introduction

While the World Health Organization (WHO) no longer considers the COVID-19 pandemic a public health emergency of international concern [1], COVID-19 remains a challenge in patients with hematological malignancies, especially those with lymphoid malignancies [2,3]. This is potentially a result of inferior serological responses to the COVID-19 vaccines in this population [2,3,4,5]. Vaccine immunity from the original mRNA-based COVID-19 vaccines wanes over time and, with the emergence of new viral variants, the efficacy of the original vaccines has been decreasing in patients with hematological malignancies as well as the general population [6]. Updated mRNA vaccines have been developed to better target emerging variants, like the nucleoside-modified bivalent mRNA vaccines Comirnaty Original/Omicron BA.1 and Comirnaty Original/Omicron BA.4-5 that are designed for the dominant endemic variants of the Omicron SARS-CoV-2 strains BA.1, and BA.4 and BA.5, respectively, in addition to the ancestral strain. Seasonally updated booster vaccinations have been offered to at-risk populations, as is already practiced with seasonally adapted vaccinations against influenza [7]. However, the effect of Omicron-adapted booster vaccination against COVID-19 in patients with lymphoid malignancies has not yet been studied. Here, we investigate the serological response to Omicron-adapted COVID-19 vaccines in patients with lymphoid malignancies and, via exploratory analyses, examine factors associated with impaired response.

## 2. Materials and Methods

### 2.1. Study Design and Participants

This study was nested in a prospective observational cohort study initiated between December 2020 and April 2021 of patients with hematological malignancies aged 18 years or older and followed at Copenhagen University Hospital, Rigshospitalet, and Herlev-Gentofte Hospital [5,8]. Patients were invited to provide blood samples between 27 December 2020 and 28 February 2023, beginning at the time of their first vaccination and up to two years after the first vaccine dose, regardless of additional vaccine doses administered. Blood sample collections were planned according to the time of the first vaccination and took place at study entry, three weeks, and two, six, twelve, eighteen, and twenty-four months after the first dose. The findings from the first five time points have been published elsewhere [5].

Patients with lymphoid malignancies were included in this sub-study if they (a) provided a blood sample at both the eighteen- and twenty-four-month time points, and (b) received an mRNA-based BNT162b2 COVID-19 vaccine with Omicron-adapted mRNA of either the BA.1 or BA.4-5 strains as their fourth or fifth COVID-19 vaccine dose between the two blood sample dates. Patients were excluded if they had received vaccine types other than BNT162b2 or if they had received more than one dose of a COVID-19 vaccine between the two sample collections. To analyze the response to the Omicron-adapted vaccines, the eighteen-month sample was considered the baseline sample for the present analysis and the twenty-four-month sample was considered the follow-up sample.

Participation in the study was voluntary and did not interfere with the vaccination strategy. COVID-19 vaccines were offered as part of the Danish vaccination program. Consent was provided by all participants after receiving oral and written information. The study was approved by the Regional Scientific Ethics Committee of the Capital Region of Denmark (H-20079890) and was conducted in accordance with the Declaration of Helsinki.

Information on diagnosis and treatment with CD20-, CD30-, or CD38-targeted antibody therapies, proteasome inhibitors, protein kinase, phosphatidylinositol-3 kinase (PI3K) or B-cell lymphoma 2 (BCL2) inhibitors, immunomodulatory drugs (IMiDs), human immunoglobulin, and SARS-CoV-2-targeted antibody therapy was collected from medical records. Confirmation of positive polymerase chain reaction (PCR) tests for SARS-CoV-2 was obtained from the Danish Microbiology Database (MiBa), and information on type and timing of COVID-19 vaccinations were collected from the Danish Vaccination Register (DDV) [9,10].

### 2.2. Antibody Quantification

An in-house ELISA based on the ancestral SARS-CoV-2 strain was used to measure IgG antibodies specific for the receptor-binding domain (RBD) of the spike (S) protein as described elsewhere [5,8]. In brief, 1 μg/mL purified recombinant RBD of the ancestral variant was coated onto Nunc Maxisorp 384-well plates (Thermo Fisher Scientific, Waltham, MA, USA) overnight in phosphate-buffered saline (PBS) (Rigshospitalet, Copenhagen, Denmark). The wells were blocked for one hour in PBS and 0.05% Tween 20 (PBS-T, Merck, Darmstadt, Germany) before adding the patient serum diluted 1:400, 1:1200, and 1:3600 in PBS-T with 5 mM EDTA and 5% skim milk. RBD-bound IgG was detected using horseradish peroxidase conjugated polyclonal rabbit-anti-human IgG (Agilent Technologies, Santa Clara, CA, USA) at a concentration of 0.5 μg/mL diluted in PBS-T. Tetramethylbenzidine (TMB) One substrate (Kem-En-Tec, Taastrup, Denmark) was added, and the reaction was stopped using H2SO4. The optical density was measured using a Synergy HT absorbance reader (BioTek Instruments, Winooski, VT, USA). Between each step, the plates were washed in PBS-T four times. IgG concentrations were calculated in Arbitrary Units (AU)/mL. The threshold of a positive IgG response was set to 225 AU/mL based on a receiver operating characteristic (ROC) curve analysis to estimate the optimal cut-off between serum from naturally infected convalescent individuals and serum from individuals obtained before 2020. Samples with a value below 1 AU/mL were set to 1 AU/mL [11].

Antibodies against the nucleocapsid (N) protein were measured using an electrochemiluminescence assay according to the manufacturer’s instructions (Elecsys^®^ Anti-SARS-CoV-2 assay, Roche Diagnostics GmbH, Mannheim, Germany). The mRNA in the BNT162b2 vaccine does not encode the N-protein. Therefore, N-antibodies are used as a marker of natural infection. Because we have previously observed low ability in producing antibodies in this specific population [5], a previous SARS-CoV-2 infection was defined on the basis of either detectable N-antibodies in the sample or a documented positive PCR test during the 12 months prior to the baseline sample.

### 2.3. Measurement of Virus-Neutralizing Capacity

As a proxy for the neutralizing capacity of the antibodies, we measured the degree of inhibition of the ACE-2 host receptor and RBD interaction using a validated in-house ELISA-based assay as previously described [8,12]. In brief, we coated 1 µg/mL in-house recombinant ACE-2 ectodomain onto Nunc Maxisorp 96-well plates in PBS overnight. A solution of patient serum diluted 1:10, Pierce High Sensitivity Streptavidin-HRP diluted 1:16,000 (Thermo Fisher Scientific), and 4 ng/mL biotinylated recombinant ancestral RBD was incubated in PBS-T in non-binding 96-well plates for one hour. The mixtures of biotinylated RBD/Streptavidin-HRP and patient serum were transferred to the ACE-2 ectodomain-coated wells and incubated for 35 min. The wells were washed three times with PBS-T between each step and developed for 20 min. The threshold for assay positivity was set to 25% inhibition in 10% diluted serum based on an ROC curve analysis to estimate the optimal cut-off between naturally infected convalescent sera and sera from individuals obtained before 2020 [11].

### 2.4. Statistics and Modeling

Continuous data were presented as medians with interquartile range (IQR) or means with standard deviation (SD). Categorical data were presented as frequency counts and percentages. The normality of data was assessed using quantile-quantile plots and scatter plots of residuals and fitted values. Observed anti-RBD IgG concentrations were log10 transformed before running the analyses and presented as log10 IgG concentrations in AU/mL. To visualize the observed anti-RBD IgG concentrations and neutralizing capacity at the two sample time points, baseline and follow-up measurements from each individual were plotted on top of a boxplot displaying the median and IQR with the mean concentrations marked with a plus sign (+). Differences in mean antibody concentrations and neutralizing capacity between the two time points were assessed using the Wilcoxon Signed-Ranks test.

To assess the change in antibody concentration and neutralizing capacity over the period of vaccination with an Omicron-adapted vaccine, we fitted a series of multivariable mixed-effects models. In all versions of the models, either log10-transformed IgG or neutralizing capacity were included as outcome variables and patient ID and time in months between sample collections were fitted as random effects (base model). Age (scaled to reflect the effect per ten-year increment), sex, type of malignancy, accumulated number of COVID-19 vaccinations (4 or 5), type of Omicron-adapted vaccine, a SARS-CoV-2 infection in the 12 months prior to baseline, and treatment with human immunoglobulin were added to the base model one at a time as individual fixed effects with months between sample collections and the individual variable included as an interaction term (partially adjusted model). In this way, we assessed whether factors had an impact on both the average value at baseline and the rate of change in the outcome over the vaccination window. A multivariable model with all individual fixed-effect variables added to the base model was performed as a sensitivity analysis with malignancy type and months between sample collections included as interaction terms (fully adjusted model). In additional sensitivity analyses, anti-CD20 therapy was included in the base model to assess the effect of B-cell-depleting treatment. Predicted estimates of log10 concentrations at baseline and changes in log10 concentrations between the sample collections were reported as log10 estimates with 95% CI. Predicted estimates of neutralizing capacity at baseline and changes in neutralizing capacity between the sample collections were reported as either percent (%) neutralizing capacity or percent point (pp) estimates with 95% CI. To visualize the effect of selected risk factors on the antibody concentrations and neutralizing capacity, predictions from the individual mixed-effect models were presented as xy plots with 95% CI. *p*-values < 0.05 were considered significant. All statistical analyses were performed in R [12] using the packages dplyr [13], lmerTest [14], and ggplot2 [15].

## 3. Results

In this study, we included 233 patients with lymphoid malignancies who received an Omicron-adapted BNT162b2 mRNA-based COVID-19 vaccine. Of these, 73 (31.3%) were diagnosed with CLL, 89 (38.2%) were diagnosed with lymphoma, and 71 (30.5%) were diagnosed with multiple myeloma or amyloidosis. The median age was 69 years [IQR 63; 75] and 116 patients (49.8%) were male. The vaccine adapted to the BA.1 strain was received by 93 patients (39.9%), while 140 patients (60.1%) received the BA.4-5 adapted vaccine. The Omicron-adapted vaccine comprised the fourth accumulated vaccine dose in 38 patients (16.3%) and the fifth accumulated vaccine dose in 195 patients (83.7%). The median time from baseline to administration of the Omicron-adapted vaccine was 0.8 months [IQR: 0.5; 1.2], and the median time from administration of the Omicron-adapted vaccine to collection of the follow-up sample was 3.7 months [3.2, 4.2]. The median time between collection of the baseline sample and the follow-up sample was 4.6 months [IQR: 4.0; 5.1]. At baseline, 129 patients (55.4%) had displayed evidence of a SARS-CoV-2 infection in the 12 months prior to baseline; an additional 28 patients (12.0%) displayed evidence of a SARS-CoV-2 infection by the time of the follow-up visit. Thirty-eight patients (16.3%) had received SARS-CoV-2-targeted antibody therapy within one year prior to the baseline sample. An additional three patients (1.3%) had received such treatment by the time of follow-up. Characteristics were similar between the different malignancy types; however, patients diagnosed with CLL were more likely to be male, and patients diagnosed with lymphoma had received fewer vaccine doses than those diagnosed with CLL or myeloma and amyloidosis. All patient characteristics, including distributions of B- and plasma-cell-targeted treatments, are listed in Table 1.

### 3.1. Anti-RBD IgG Concentrations

**Figure 1:**
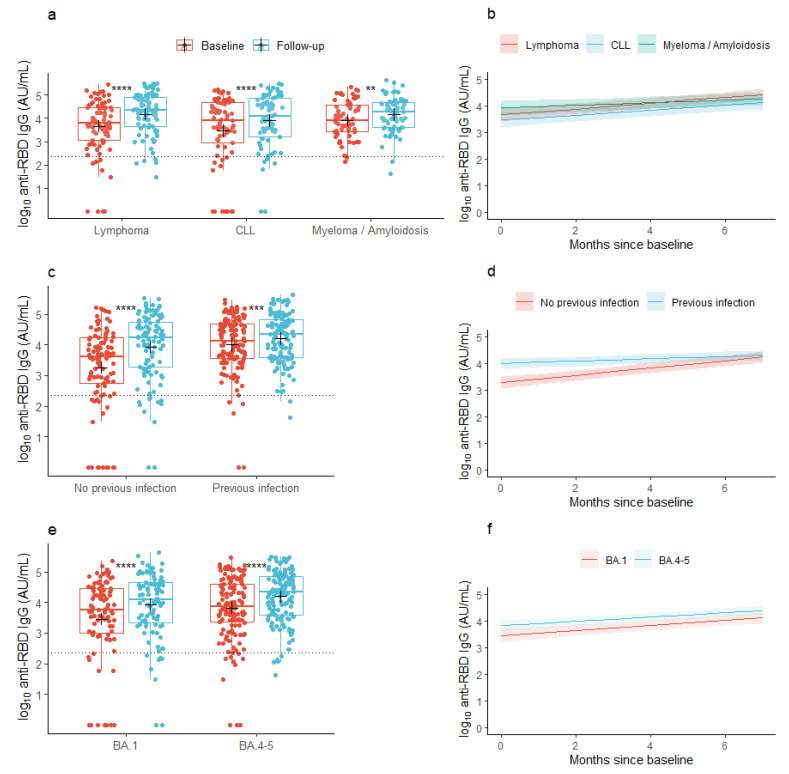
Observed and predicted anti-SARS-CoV-2 RBD concentrations. (**a**,**c**,**e**) Box plots show median concentrations of anti-RBD IgG with interquartile ranges in patients with lymphoid malignancies before and after vaccination with an Omicron-adapted mRNA-based SARS-CoV-2 vaccine plotted on top of individually observed measurements. Red: Baseline measurements. Blue: Follow-up measurements. The dotted horizontal lines indicate the minimum threshold of an IgG response. The plus sign (+) indicates the geometric mean. **: *p* < 0.01, ***: *p* < 0.001, ****: *p* < 0.0001. (**b**,**d**,**f**) Plots represent the predicted change per month in mean concentrations of anti-RBD IgG between baseline and follow-up in patients with lymphoid malignancies as predicted using linear mixed models. Shaded areas indicate 95% confidence intervals.

At baseline, the mean concentration of anti-RBD IgG was 3.67 log10 AU/mL (95% CI: 3.52; 3.83) across all the lymphoid malignancies. At follow-up, the mean concentration had increased to 4.08 log10 AU/mL (95% CI: 3.96; 4.21), *p* < 0.001), with similar changes seen in the individual malignancy groups (Figure 1a). In patients with a previous infection, the mean anti-RBD IgG concentration at baseline was 4.01 log10 AU/mL (95% CI: 3.85; 4.17) and increased to 4.20 log10 AU/mL (95% CI: 4.07; 4.34) at follow-up, while patients without a previous infection at baseline increased their anti-RBD IgG concentrations from 3.26 log10 AU/mL (95% CI: 2.99; 3.53) to 3.94 log10 AU/mL (95% CI: 3.72; 4.15) from baseline to follow-up, respectively (Figure 1c). In patients who received the BA.1-adapted vaccine, the baseline anti-RBD IgG concentration was 3.46 log10 AU/mL (95% CI: 3.16; 3.75) and increased to 3.92 log10 AU/mL (95% CI: 3.69; 4.14), while patients who received the BA.4-5-adapted vaccine increased their anti-RBD IgG concentration from 3.82 log10 AU/mL (95% CI: 3.65; 3.99) to 4.19 log10 AU/mL (95% CI: 4.06; 4.33) (Figure 1e).

Across all patients with lymphoid malignancies (the base model), the predicted mean concentration of anti-RBD IgG was 3.69 log10 AU/mL (95% CI: 3.53; 3.84) at baseline, prior to vaccination with an Omicron-adapted vaccine, and increased by 0.09 log10 AU/mL/month (95% CI: 0.07; 0.11) between the two sample time points. With lymphoma set as the reference group, the predicted mean concentration at baseline did not vary between lymphoid malignancy types (Table 2a). However, the rate of the increase in anti-RBD IgG concentration between the sample collections was lower in patients with multiple myeloma or amyloidosis compared to patients with lymphoma in both partially (−0.06 log10 AU/mL/month (95% CI: −0.11; −0.01)) and fully adjusted models (−0.06 log10 AU/mL/month (95% CI: −0.11; −0.01), Figure 1b, Table 2a). Older age was significantly associated with lower baseline concentrations, but not with the increment of the antibody concentration over the period of Omicron-adapted vaccination in partially adjusted models. In the fully adjusted model, age was not associated with the baseline antibody concentration. Neither sex nor accumulated number of vaccine doses were associated with the baseline or increment in antibody concentrations in any of the partially or fully adjusted models (Table 2a). In both partially and fully adjusted models, patients who were given the BA.4-5-adapted vaccine had higher IgG concentrations at baseline, prior to receiving the Omicron-adapted vaccine, compared to patients who were offered the BA.1-adapted vaccine; however, there was no association between the type of Omicron-adapted vaccine and the increment in antibody concentrations from baseline to follow-up (Figure 1e,f, Table 2a). A documented SARS-CoV-2 infection in the 12 months prior to baseline was associated with a higher predicted mean concentration of anti-RBD IgG at baseline in both partially and fully adjusted models and with a lower predicted increment in mean concentration of anti-RBD IgG in the partially adjusted model (−0.09 log10 AU/mL/month (95% CI: −0.135; −0.054), Figure 1c,d, Table 2a). Treatment with human immunoglobulin from one year prior to baseline was associated with lower anti-RBD IgG concentrations at baseline but not with the increment in anti-RBD IgG concentrations between the baseline sample and follow-up (Table 2a). In a sensitivity analysis, anti-CD20 treatment up to one year prior to baseline was associated with a lower predicted anti-RBD IgG concentration at baseline (−1.36 log10 AU/mL (95% CI: −2.15; −0.58)), but not with the increment in anti-RBD IgG concentrations between the sample collections (−0.01 log10 AU/mL/month (95% CI: −0.12; 0.09)).

### 3.2. Neutralizing Capacity

**Figure 2:**
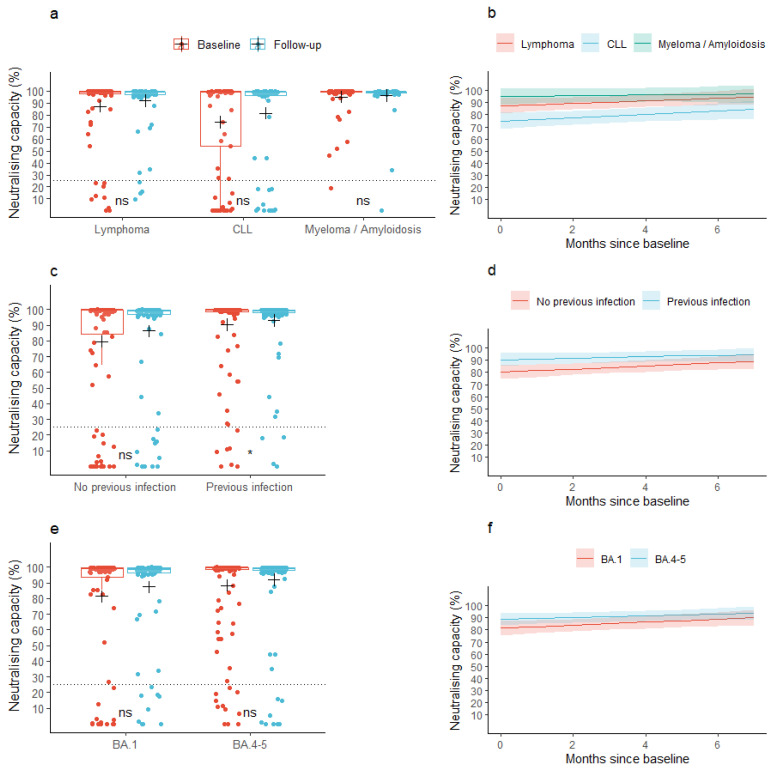
Observed and predicted neutralizing capacity. (**a**,**c**,**e**) Box plots show median neutralizing capacity with interquartile ranges in patients with lymphoid malignancies before and after vaccination with an Omicron-adapted mRNA-based SARS-CoV-2 vaccine plotted on top of individually observed measurements. Red: Baseline measurements. Blue: Follow-up measurements. The dotted horizontal lines indicate the minimum threshold for a neutralizing response. The plus sign (+) indicates the mean. ns: *p* > 0.05, *: *p* < 0.05. (**b**,**d**,**f**) Plots represent the change per month in mean neutralizing capacity between baseline and follow-up in patients with lymphoid malignancies as predicted using linear mixed models. Shaded areas indicate 95% confidence intervals.

At baseline, the mean neutralizing capacity was 85.6% (95% CI: 81.6; 89.6) across all the lymphoid malignancies. At follow-up, the mean neutralizing capacity was 90.2% (95% CI: 86.9; 93.4), *p* = 0.077). A similar pattern was observed for the individual malignancy groups (Figure 2a). In patients with a previous infection, the mean neutralizing capacity at baseline was 90.4% (95% CI: 86.2; 94.6) and increased to 93.2% (95% CI: 89.7; 96.7) at follow-up, while patients without a previous infection did not increase their neutralizing capacity significantly between the baseline and follow-up samples (neutralizing capacity: 79.7% (95% CI: 72.6; 86.7) and 86.4% (95% CI: 80.5; 92.3) at baseline and follow-up, respectively, Figure 2c). We found no change in mean neutralizing capacity from baseline to follow-up when stratifying according to the type of Omicron-adapted vaccine (Figure 2e).

When assessing the neutralizing capacity across lymphoid malignancies (the base model), the predicted mean neutralizing capacity was 85.9% (95% CI: 82.0; 89.8) at baseline, prior to vaccination with an Omicron-adapted vaccine, and increased by 0.9 pp/month (95% CI: 0.2; 1.6) between the two sample time points. The predicted mean neutralizing capacity at baseline was significantly lower in patients diagnosed with CLL compared to patients diagnosed with lymphoma (partially adjusted model: −12.4 pp (95% CI: −21.4; −3.4), fully adjusted model: −12.0 pp (95% CI: −21.0; −3.0)), but not compared to patients diagnosed with myeloma or amyloidosis (Figure 2b, Table 2b). We found no difference in the rate with which the neutralizing capacity increased between the lymphoid malignancy types in neither the partially nor fully adjusted models (Figure 2b, Table 2b). Age, sex, accumulated number of vaccine doses, or type of Omicron-adapted vaccine were not associated with baseline neutralizing capacity or an increase in neutralizing capacity in either of the partially or fully adjusted models (Table 2b). Having been infected with SARS-CoV-2 less than one year prior to the baseline sample was associated with higher predicted mean neutralizing capacity at baseline in both the partially and fully adjusted models, but not with a change in neutralizing capacity between the two sample collections. Treatment with human immunoglobulin was not associated with the neutralizing capacity at baseline nor with an increase in neutralizing capacity (Table 2b). In a sensitivity analysis, anti-CD20 treatment up to one year prior to collection of the baseline sample was associated with a lower predicted neutralizing capacity at baseline (−21.2 pp (95% CI: −41.2; −1.2)) and with a lower increase in neutralizing capacity over the period of Omicron-adapted vaccination (−6.4 pp (95% CI: −9.9; −2.8)).

## 4. Discussion

Here, we studied antibody concentrations and neutralizing capacity over the period of booster vaccination with a bivalent Omicron-adapted BNT162b2 COVID-19 vaccine in patients with lymphoid malignancies. We found increases in concentrations of anti-RBD IgG and neutralizing capacity over the period of vaccination with an Omicron-adapted booster vaccine across lymphoid malignancies, regardless of the variant of the adapted vaccine, although the increase in neutralizing capacity was rather discrete. The increase in anti-RBD IgG between the sample collections was lower in patients with multiple myeloma or amyloidosis compared to patients with lymphoma, while no difference was seen between patients with CLL and lymphoma. Patients who had been infected with SARS-CoV-2 within one year prior to baseline had higher baseline antibody concentrations than those who had not contracted a SARS-CoV-2 infection in the same period. However, the increase in antibody concentration after receiving the booster vaccine was less pronounced in patients with a previous infection. A similar pattern was seen for the neutralizing capacity, although the change in neutralizing capacity between the two sample collections was not statistically associated with a previous infection with SARS-CoV-2 up to one year prior to the baseline.

When contemplating the introduction of modified COVID-19 vaccines for seasonal vaccination schemes, similar to the approach taken with the influenza vaccine, numerous factors should be taken into account, including the speed of virus mutations and the timing of the highest infection rates during the year. Immune imprinting, the phenomenon where previous exposure to a pathogen affects the production of antibodies targeting new variants of the same pathogen when encountering these, has been raised as an additional challenge to seasonal COVID-19 vaccination, as antibodies induced by the first encountered variants might outweigh antibodies produced in response to new variants when the immune system encounters these, even if these are introduced via mRNA-based vaccines [16,17,18]. However, the improved neutralization of antigenic divergent variants has been seen after variant-adapted booster vaccination [19].

Here, we found higher baseline antibody concentrations and neutralizing capacity in patients with a previous SARS-CoV-2 infection at baseline than in patients without a previous infection, but with a lower increase in antibody concentration after receiving the booster vaccine in patients with a previous infection. Similar patterns were seen in a recent study investigating the development of antibodies specific to both ancestral and Omicron variants BA.1 through BA.5 in Danish citizens above 50 years of age or at increased risk of severe COVID-19 [20].

In our data, patients who received the BA.4-5-adapted vaccine presented with higher antibody concentrations at baseline than those who received the BA.1-adapted vaccine while the type of variant-adapted vaccine was not associated with either the baseline neutralizing capacity or changes in neutralizing capacity over the vaccination period. The discrepancy in antibody concentrations is possibly attributable to a timing bias in vaccine availability. Given that the BA.1-adapted vaccine was released before the BA.4-5-adapted vaccine was approved by the European Medicines Agency (EMA), it is conceivable that individuals who were initially at higher risk of poor immune responses, who had initially displayed weaker antibody responses to their primary vaccinations, or who had not recently been exposed to infection, might have been more inclined to seek an early booster vaccination [21]. The lower increase in anti-RBD IgG in patients with multiple myeloma or amyloidosis may additionally be explained by a higher antibody concentration at baseline in this population as an expression of antibody homeostasis and peak antibody production.

Consistent with previous observations of neutralizing capacity after three or four doses of the original BNT162b2 COVID-19 vaccine in the same cohort, the predicted mean neutralizing capacity at baseline was significantly lower in patients diagnosed with CLL compared to patients diagnosed with lymphoma in this study; however, the increment in antibody concentration was not affected by the type of malignancy [5].

Our study has some limitations. The study design was based on the original two-dose regimen and evaluated humoral immune responses against the ancestral RBD antigen of SARS-CoV-2, despite Omicron being the dominant strain when the Omicron-adapted vaccines were offered. The timing and number of samples collected did not allow for dynamic modeling of the antibody concentrations over the vaccination period; however, the linear model was applied in a pragmatic approach to evaluate the development of antibodies over the course of Omicron-adapted vaccination. Additionally, we did not have data on symptom presentation or hospitalization among infected individuals, which prevented us from correlating immune responses with disease severity, and the lack of data on variant-adapted booster vaccination in healthy individuals prevented a comparison with the background population. Furthermore, our study lacked the power to assess protection against infection. The strengths of our study lie in the relatively large study population, the extensive follow-up period, and information on Omicron-adapted vaccine types.

In conclusion, antibody concentrations and neutralizing capacity increased over the period of Omicron-adapted vaccination in patients with lymphoid malignancies regardless of the adapted vaccine variant and type of lymphoid malignancy; thus, patients with lymphoid malignancies are likely to benefit from Omicron-adapted booster vaccination.

## Figures and Tables

**Table 1:** Participant characteristics.

		All Patients n = 233	Chronic Lymphatic B-Cell Leukemia n = 73 (31.3%)	Lymphoma n = 89 (38.2%)	Multiple Myeloma/Amyloidosis n = 71 (30.5%)	*p*-Value
Age; years, median [IQR]		69 [63, 75]	71 [66, 75]	68 [62, 74]	69 [62, 76]	0.172
Male sex, n (%)		116 (49.8)	46 (63.0)	40 (44.9)	30 (42.3)	0.023
BMI, mean (SD)		25.4 (4.1) Missing: 5	25.3 (4.1) Missing: 2	25.6 (4) Missing: 2	25.4 (4.1) Missing: 1	0.739
No. of COVID-19 vaccines at follow-up, n (%)	4 doses, n (%)	38 (16.3)	13 (17.8)	21 (23.6)	4 (5.6)	-
	5 doses, n (%)	195 (83.7)	60 (82.2)	68 (76.4)	67 (94.4)	0.009
Vaccine variant	BA. 1	93 (39.9)	28 (38.4)	37 (41.6)	28 (39.4)	-
	BA. 4-5	140 (60.1)	45 (61.6)	52 (58.4)	43 (60.6)	0.913
Months between samples, median [IQR]		4.6 [4.0, 5.1]	4.4 [4.1, 5.0]	4.5 [4.1, 5.1]	4.6 [3.9, 5.1]	0.820
Months from baseline to vaccination, median [IQR]		0.8 [0.5, 1.2]	0.7 [0.3, 1.2]	0.8 [0.5, 1.2]	0.9 [0.5, 1.2]	0.513
Months from vaccination to follow-up, median [IQR]		3.7 [3.2, 4.2]	3.8 [3.3, 4.1]	3.8 [3.2, 4.2]	3.6 [3.0, 4.1]	0.237
Days between doses, median [IQR]	1st to 2nd dose	23 [21, 24]	23 [21, 25]	23 [22, 24]	23 [21, 24]	0.372
	2nd to 3rd dose	183 [175, 203]	179 [172, 202]	184 [176, 212]	184 [176, 201]	0.150
	3rd to 4th dose	141 [136, 179]	142 [137, 179]	141 [138, 285]	140 [133, 147.5]	0.012
	4th to 5th dose	248 [238, 254.5]	248 [242, 254.2]	248 [240.5, 252]	249 [237, 257]	0.737
COVID-19 infection (PCR or N-Ab), n (%)	At baseline	129 (55.4)	40 (54.8)	46 (51.7)	43 (60.6)	0.529
	At follow-up	157 (67.4)	49 (67.1)	57 (64.0)	51 (71.8)	0.579
Treatment with anti-SARS-CoV-2 immunoglobulins	<1 year prior to baseline	38 (16.3)	16 (21.9)	8 (9.0)	14 (19.7)	0.056
	By time of follow-up	41 (17.6)	17 (23.3)	9 (10.1)	15 (21.1)	0.058
Treatment with human immunoglobulin	<1 year prior to baseline	26 (11.2)	12 (16.4)	7 (7.9)	7 (9.9)	0.207
	By time of follow-up	32 (13.7)	12 (16.4)	10 (11.2)	10 (14.1)	0.629
B-cell- or plasma-cell-targeted treatment and immunomodulatory treatment, n (% of patient population) <1 year prior to baseline	Anti-CD20 therapy	9 (3.9)	3 (4.1)	6 (6.7)	-	0.088
	Anti-CD30 therapy	1 (0.4)	-	1 (1.1)	-	0.444
	Anti-CD38 therapy	25 (10.7)	-	-	25 (35.2)	<0.001
	Proteasome inhibitors	7 (3.0)	-	-	7 (9.9)	<0.001
	Proteinkinase, PI3K, or BCL2 inhibitors	24 (10.3)	17 (23.3)	6 (6.7)	1 (1.4)	<0.001
	Immunomodulatory imide drugs (IMiDs)	25 (10.7)	-	1 (1.1)	24 (33.8)	<0.001

**Table 2:** Differences in SARS-CoV-2 RBD IgG (a) and neutralizing capacity (b) between potential risk groups.

(a)		SARS-CoV-2 anti-RBD IgG Partially Adjusted Model *				SARS-CoV-2 anti-RBD IgG Fully Adjusted Model ^#^			
		Difference in Baseline log_10_ AU/mL (95% CI)	*p*-Value	Change in log_10_ AU/mL/Month (95% CI)	*p*-Value	Difference in Baseline log_10_ AU/mL (95% CI)	*p*-Value	Change in log_10_ AU/mL/Month (95% CI)	*p*-Value
Malignancy type	Lymphoma	Ref	Ref	Ref	Ref	Ref	Ref	Ref	Ref
	Myeloma/Amyloidosis	0.255 (−0.117, 0.627)	0.181	−0.059 (−0.109; −0.009)	0.022	0.267 (−0.088; 0.623)	0.147	−0.058 (−0.108; −0.008)	0.025
	CLL	−0.198 (−0.567; 0.171)	0.295	−0.017 (−0.066; 0.033)	0.515	−0.159 (−0.513; 0.195)	0.385	−0.014 (−0.064; 0.036)	0.573
Age, per 10 years		−0.209 (−0.378; −0.040)	0.016	0.018 (−0.005; 0.041)	0.120	−0.067 (−0.203; 0.070)	0.345	-	-
Sex	Female	Ref	Ref	Ref	Ref	Ref	Ref	-	-
	Male	0.057 (−0.253; 0.366)	0.721	−0.009 (−0.051; 0.033)	0.680	0.067 (−0.173; 0.308)	0.592	-	-
No. of COVID-19 vaccines at follow-up	4 doses	Ref	Ref	Ref	Ref	Ref	Ref	-	-
	5 doses	−0.159 (−0.578; 0.259)	0.456	−0.027 (−0.085; 0.030)	0.355	−0.197 (−0.526; 0.132)	0.249	-	-
Vaccine variant	BA.1	Ref	Ref	Ref	Ref	Ref	Ref	-	-
	BA.4-5	0.363 (0.051; 0.675)	0.024	−0.012 (−0.055; −0.030)	0.566	0.252 (0.004; 0.500)	0.052	-	-
COVID-19 infection (verified using PCR and/or N-Ab) < 1 year prior to baseline		0.723 (0.426; 1.020)	<0.001	−0.094 (−0.135; −0.054)	< 0.001	0.358 (0.103; 0.612)	0.005	-	-
Treatment with human immunoglobulin < 1 year prior to baseline		−0.494 (−0.982; −0.007)	0.048	0.016 (−0.052; 0.083)	0.644	−0.498 (−0.884; −0.111)	0.013	-	-
**(b)**		**Neutralizing Capacity** Partially Adjusted Model *				**Neutralizing Capacity** **Fully Adjusted Model ^#^**			
		**Difference in Baseline% (95% CI)**	***p*-Value**	**Change in pp/Month (95% CI)**	***p*-Value**	**Difference in Baseline% (95% CI)**	***p*-Value**	**Change in pp/Month (95% CI)**	***p*-Value**
Malignancy type	Lymphoma	Ref	Ref	Ref	Ref	Ref	Ref	Ref	Ref
	Myeloma/Amyloidosis	7.8 (−1.3; 16.9)	0.094	−0.8 (−2.5; 1.0)	0.388	−8.4 (−0.6; 17.4)	0.073	−0.8 (−2.5; 1.0)	0.389
	CLL	−12.4 (−21.4; −3.4)	0.008	0.3 (−1.4; 2.0)	0.751	−12.0 (−21.0; −3.0)	0.010	0.3 (−1.4; 2.0)	0.708
Age, per 10 years		−4.2 (−8.4; 0.1)	0.057	0.2 (−0.5; 1.0)	0.540	−1.5 (−4.9; 1.9)	0.403	-	-
Sex	Female	Ref	Ref	Ref	Ref	Ref	Ref	-	-
	Male	−1.5 (−9.2; 6.3)	0.715	−0.5 (−1.9; 1.0	0.524	−0.5 (−6.5; 5.5)	0.874	-	-
No. of COVID-19 vaccines at follow-up	4 doses	Ref	Ref	Ref	Ref	Ref	Ref	-	-
	5 doses	−7.5 (−18.0; 2.9)	0.160	0.6 (−1.4; 2.6)	0.546	−6.5 (−14.7; 1.8)	0.130	-	-
Vaccine variant	BA.1	Ref	Ref	Ref	Ref	Ref	Ref	-	-
	BA.4-5	7.1 (−0.7; 15.0)	0.078	−0.5 (−1.9; 1.0)	0.512	4.2 (−2.0; 10.5)	0.190	-	-
COVID-19 infection (verified using PCR and/or N-Ab) < 1 year prior to baseline		10.3 (2.6; 18.0)	0.010	−0.7 (−2.1; 0.8)	0.357	6.7 (0.6; 12.8)	0.035	-	-
Treatment with human immunoglobulin < 1 year prior to baseline		−5.5 (−17.8; 6.8)	0.385	1.9 (−0.4; 4.1)	0.108	0.3 (−9.4; 10.0)	0.954	-	-

*: Model included patient ID and time in months between sample collections as random effects. Age (scaled to reflect the effect per ten-year increment), sex, type of malignancy, accumulated number of COVID-19 vaccinations (4 or 5), type of Omicron-adapted vaccine, a SARS-CoV-2 infection in the 12 months prior to baseline, and treatment with human immunoglobulin were added to the base model one at a time as individual fixed effects with months between sample collections and the individual variable included as an interaction term. ^#^: Model included patient ID and time in months between sample collections as random effects. All individual fixed-effect variables listed under the partially adjusted model were included in one model with malignancy type and months between sample collections included as interaction term. -: Not assessed.

## Data Availability

The datasets generated and analyzed during the current study are available in de-identified format from the corresponding author on reasonable request.
